# Disrupting SARS-CoV-2 Spike Protein Activity: A Virtual Screening and Binding Assay Study

**DOI:** 10.3390/ijms26010151

**Published:** 2024-12-27

**Authors:** Luís Queirós-Reis, Rui Alvites, Ana Colette Maurício, Andrea Brancale, Marcella Bassetto, João R. Mesquita

**Affiliations:** 1 Abel Salazar Institute of Biomedical Sciences (ICBAS), University of Porto, 4050-313 Porto, Portugal; up201205115@up.pt (L.Q.-R.); rmalvites@icbas.up.pt (R.A.); acmauricio@icbas.up.pt (A.C.M.); 2 Animal Science Study Centre (CECA), University of Porto Agroenvironment, Technologies and Sciences Institute (ICETA), 4051-401 Porto, Portugal; 3 Associate Laboratory for Animal and Veterinary Science (AL4AnimalS), 1300-477 Lisboa, Portugal; 4 Advanced Polytechnic and University Cooperative, University Institute of Health Sciences (CESPU), Avenida Central de Gandra 1317, 4585-116 Gandra, Portugal; 5 Department of Organic Chemistry, University of Chemistry and Technology Prague, 166 28 Prague, Czech Republic; andrea.brancale@vscht.cz; 6 School of Pharmacy and Pharmaceutical Sciences, College of Biomedical and Life Sciences, Cardiff University, Cardiff CF10 3BN, UK; bassettom1@cardiff.ac.uk; 7 Department of Chemistry, Faculty of Science and Engineering, Swansea University, Swansea SA2 8PP, UK; 8 Epidemiology Research Unit (EPIunit), Institute of Public Health, University of Porto, 4050-091 Porto, Portugal

**Keywords:** SARS-CoV-2, spike glycoprotein, virtual screening, in vitro assays

## Abstract

Severe Acute Respiratory Syndrome Coronavirus 2 (SARS-CoV-2) is a respiratory virus that emerged in late 2019 and rapidly spread worldwide, causing the COVID-19 pandemic. The spike glycoprotein (S protein) plays a crucial role in viral target recognition and entry by interacting with angiotensin, converting enzyme 2 (ACE2), the functional receptor for the virus, via its receptor binding domain (RBD). The RBD availability for this interaction can be influenced by external factors, such as fatty acids. Linoleic acid (LA), a free fatty acid, has been shown to bind the S protein, modulating the viral infection by reducing initial target recognition. LA interacts with the fatty acid binding pocket (FABP), a potential drug target against SARS-CoV-2. In this study, we aimed to exploit the FABP as a drug target by performing a docking-based virtual screening with a library of commercially available, drug-like compounds. The virtual hits identified were then assessed in in vitro assays for the inhibition of the virus–host interaction and cytotoxicity. Binding assays targeting the spike–ACE2 interaction identified multiple compounds with inhibitory activity and low cytotoxicity.

## 1. Introduction

In late 2019, a new viral infection was detected in Wuhan, China, caused by a new coronavirus (CoV), Severe Acute Respiratory Syndrome Coronavirus 2 (SARS-CoV-2). This virus rapidly spread worldwide in a few months, leading to the COVID-19 pandemic, infecting more than 750 million people and causing over seven million deaths [1]. SARS-CoV-2 is an RNA virus, belonging to the Coronaviridae family, originated in bats [2,3,4].

However, the course of the pandemic was changed by the introduction of vaccines against SARS-CoV-2 [5]. In fact, the recent development of vaccines was considered a powerful measure to save lives and minimize the impact on health, social systems, and global economics [6]. It is well known that SARS-CoV-2 genome mutations influence the efficacy of the immune response induced by vaccination [5]. Since the beginning of the COVID-19 pandemic, numerous mutations of SARS-CoV-2 have been identified [5]. Periodic viral genomic sequencing helps to detect new genetic variants circulating in communities [5]. An updated version of the SARS-CoV-2 phylogenetic tree is shared on the GISAID platform (Global Initiative on Sharing Avian Influenza Data). A variant is recognized as a Variant of Concern (VOC) or Variant of Interest (VOI) by the World Health Organization (WHO) [5].

**Figure 1:**
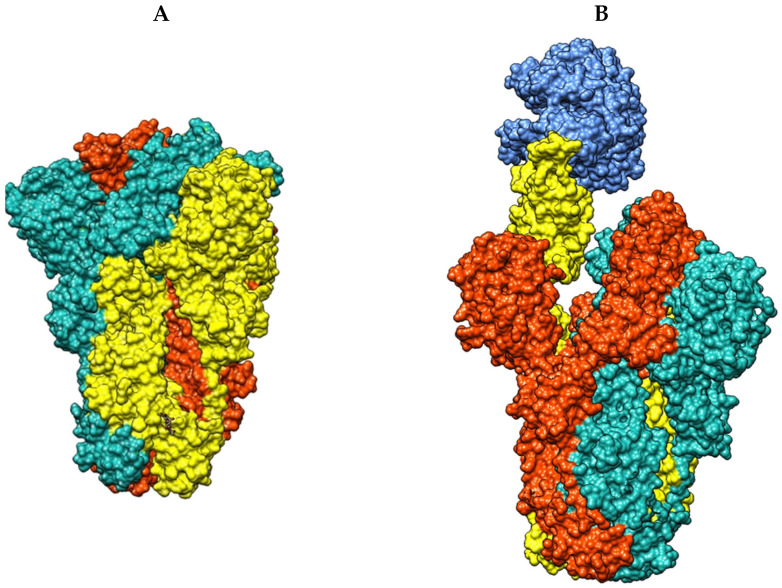
(**A**) Protein surface representation of the S trimer in a three RBD down conformation (blue, orange, yellow) (PDB ID: 7KMS). (**B**) Protein surface representation of the S trimer in a one RBD up (yellow) bound to ACE2 (dark blue), and two RBDs down (blue and orange) conformation (PDB ID: 8IOU).

As overall observed in CoVs, cell entry is highly dependent on the spike glycoprotein (S protein), a structural surface protein, which recognizes the human angiotensin converting enzyme 2 (ACE2) [3,7]. The S protein is a trimeric fusion protein fully responsible for cell recognition and cell entry and is thus a main target for neutralizing antibodies, as well as therapeutics and vaccines [2,3]. The key area of interaction in each monomer of the S protein is the receptor binding domain (RBD) that interacts with ACE2 and initiates cell infection [7]. However, the RBDs have a dynamic nature, showing two conformations: a down conformation (inactive), inaccessible for interaction with ACE2, and an up conformation (active), required for target recognition, as well as neutralizing antibodies [3,8,9,10]. Each RBD can individually change between conformations, and the availability of RBDs can be influenced by external factors, such as the pH or the presence of ACE2 in the medium, as each binding event to ACE2 promotes further RBD change to the active conformation in the same S protein trimer (Figure 1) [8,11,12].

Another external factor that can affect RBD accessibility to ACE2 recognition is the presence of free fatty acids (FFAs), since these affect the balance of open/closed RBDs. FFAs, such as linoleic acid (LA), are essential eicosanoid precursors and tissue inflammatory regulators [13], whose concentration is severely increased during infection and lung inflammation states [14]. LA affects the S protein and RBD behavior by binding a site within the protein, the fatty acid binding pocket (FABP) [14,15]. Physiologically, FFAs are proposed to function as molecular switches, enabling SARS-CoV-2 to adapt its immunogenicity to inflammatory state by changing from a high infective status before a host immune response is established, to a reduced viral recognition and clearance state, increasing viral titers in inflammatory states [14]. Pharmacologically, this mechanism might be exploited by using compounds mimicking LA to permanently stabilize the S protein in an inactive conformation [15].

**Figure 2:**
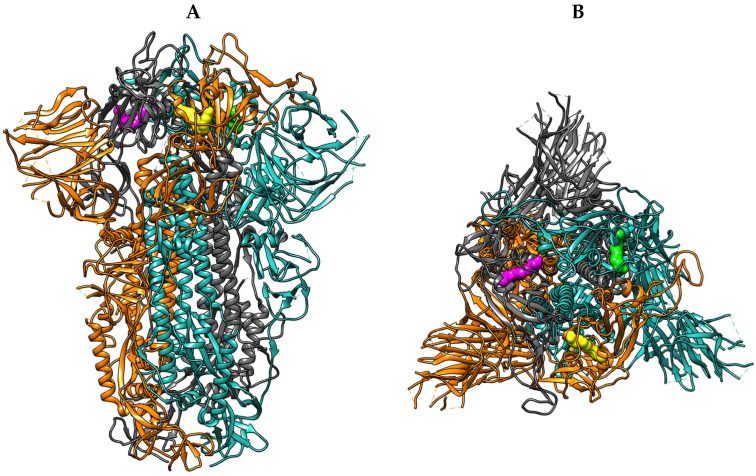
(**A**) Side view of the S protein in ribbon representation with the monomers represented as blue, red, and grey ribbons, along with the three FABPs in yellow, green, and magenta molecular surfaces (PBD ID: 6ZB5). (**B**) Top view of the S protein in ribbon representation with the monomers represented as blue, orange, and grey ribbons, along with the three FABPs in yellow, green, and magenta molecular surfaces (PBD ID: 6ZB5).

When LA is bound to the FABP, the stabilized inactive conformation hides the receptor binding motifs (RBM) between RBD interfaces, preventing interaction with ACE2, thereby reducing virus–host interactions, cellular recognition, and infection [15]. The FABP is formed between RBD pairs, being independent of the RBM. This structural arrangement results in three similar FABPs detected between S protein monomers (Figure 2) [15].

**Figure 3:**
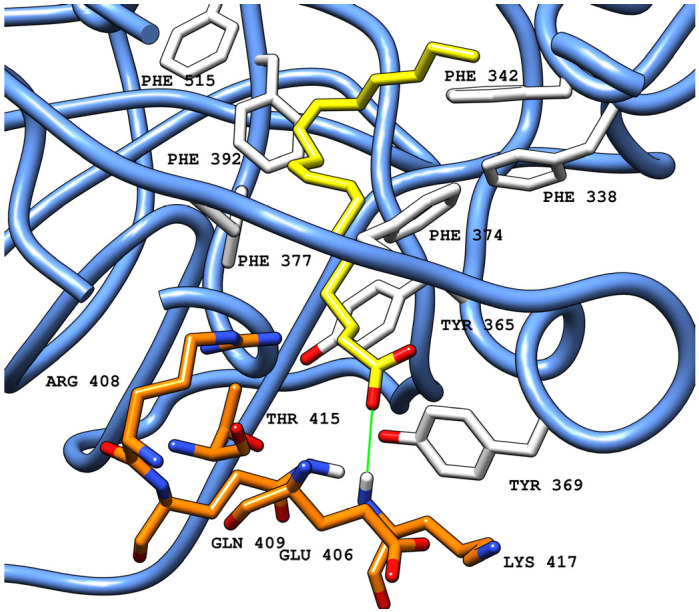
Crystallized LA (PDB ID: 6ZB5) (carbon atoms in yellow) in the FABP in a ribbon representation (blue), located between two adjacent RBDs (carbon atoms in white from RBD-B and carbon atoms in orange from RBD-C). Green solid lines represent polar interactions (hydrogen bonds) between the ligand and amino acid residues in the protein. All red atoms corresponds to oxygen atoms, while grey is due to shadows in the picture for depth perception.

Structurally, the FABP features a bent hydrophobic pocket and a hydrophilic entrance. The hydrophobic pocket is lined with multiple phenylalanine residues from one RBD, while the entrance is formed by hydrophilic residues from the adjacent RBD. Each FABP can be divided into three main interaction areas: (1) a deep hydrophobic region, primarily formed by phenylalanine residues; (2) an intermediate region, characterized by phenylalanine and tyrosine residues that act as gating helices (Tyr365 and Tyr369); and (3) a hydrophilic entrance, with multiple residues from the adjacent RBD (Arg408, Gln409) [16]. LA can establish multiple interactions across the pocket, with the hydrocarbon chain interacting in deeper regions, while the carboxylic tail establishes electrostatic interactions with residues in the intermediate and entrance areas, locking the ligand in the final conformation (Figure 3) [15].

Overall, the FABP can affect the S protein and RBM exposure, although it does not interact directly, and therefore is not affected by the high frequency of mutations in this area of the protein [17]. On the other hand, the FABP can be detected in all SARS-CoV-2 variants, as well as in other highly pathogenic coronavirus [17].

In this study, we aimed to explore the biological effect of FFAs, shown by LA binding, using the FABP as a drug target to affect the virus lifecycle. A compound capable of binding the FABP and mimicking the effects of LA could significantly reduce the virus’s ability to infect new cells, regardless of inflammatory state. The physiological role of the FABP may also present an additional barrier to resistance development, while the high conservation of this site in other highly pathogenic coronaviruses positions the FABP as a potential broad-spectrum anti-CoV target. To achieve this, a docking-based virtual screening was performed of a library of commercial, drug-like compounds, against the crystal structure of LA bound to the FABP. The virtual hits identified were then assessed in in vitro assays for the inhibition of the virus–host interaction and cytotoxicity.

## 2. Results

### 2.1. Identification of Compounds

**Figure 4:**
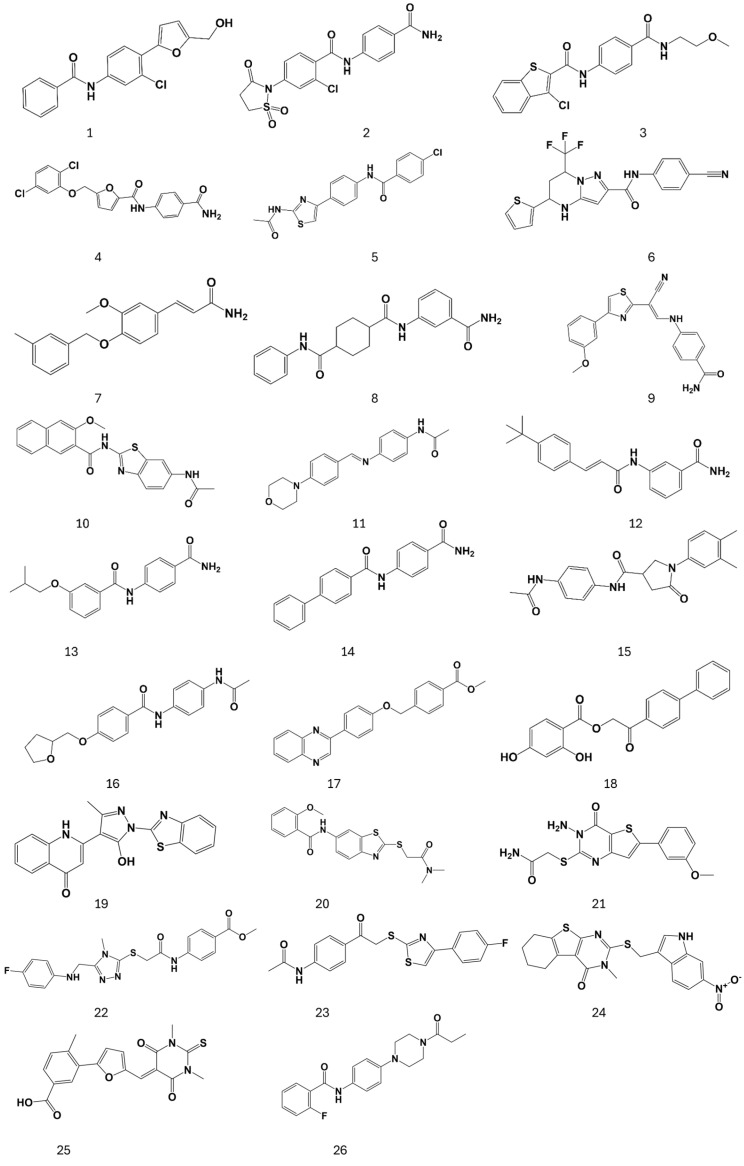
Chemical structure of compounds selected after the structure-based virtual screening and purchased from SPECS, identifying each compound with a number (1-26).

To explore the FABP effects on the S protein, particularly the stabilization of an inactive conformation by a small molecule, the SPECS library of over 350,000 drug-like compounds was screened against the three FABPs in the crystal structure of LA bound to the S protein [15]. The virtual screening was performed with the Glide Standard Precision (SP) docking tool [18], with docking poses generated for each compound in each FABP, using LA molecules as centroids from SARS-CoV-2 Wuhan S protein (PDB ID: 6ZB5) [15]. Docking poses were then rescored with three scoring functions: Glide XP, CHEMPLP (PLANTS), and OpenEye (ScorePose) [19,20,21]. After applying an in-house optimized consensus scoring procedure [22], docking poses falling in the top 25% of the score range for all scoring functions were selected for visual inspection. A total of 5000 molecules for each binding site were chosen for visual inspection and combined into one set containing molecules common in the three selection sets. The selection of molecules was further reduced according to the predicted interactions, pharmacokinetics, and drug-like properties, resulting in 26 molecules (Figure 4). The final selection was purchased from SPECS and evaluated in S protein–ACE2 binding inhibition assays. Predicted poses shown are from the FABP formed by RBD-B and RBD-A.

**Figure 5:**
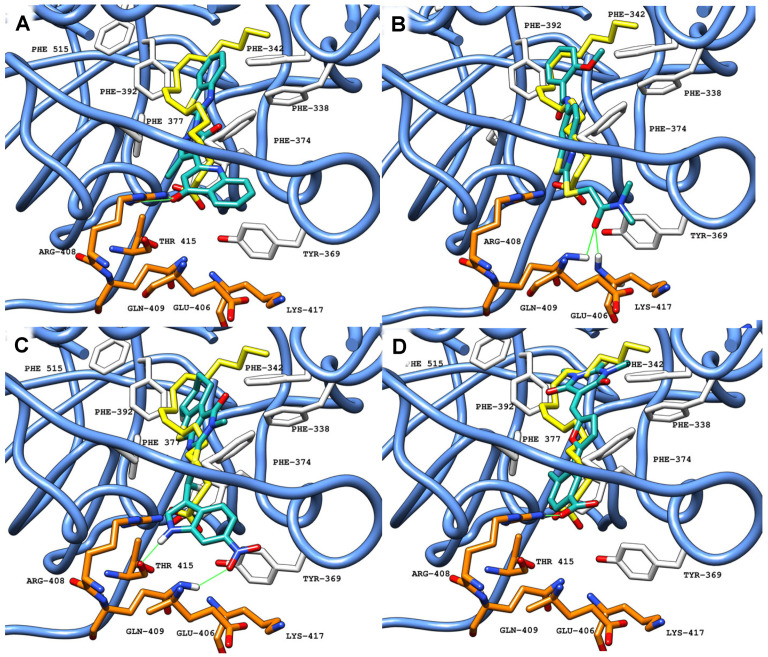
Molecular docking results obtained for **19** (**A**), **20** (**B**), **24** (**C**), and **25** (**D**), represented with carbon atoms in sea green, in the FABP of SARS-CoV-2 S protein (PDB ID: 6ZB5, blue ribbon, carbon atoms in white from RBD-1 and orange from RBD-2). Green solid lines represent polar interactions (hydrogen bonds) between the ligand and amino acid residues in the protein.

Overall, the presence of aromatic rings is the main feature observed in the selected compounds, along with highly hydrophilic groups in at least one terminal end of the molecule. Four representative compounds **19**, **20**, **24,** and **25** are shown (Figure 5), having the best activity profile among the screened compounds in our assays, detailed below.

**Figure 6:**
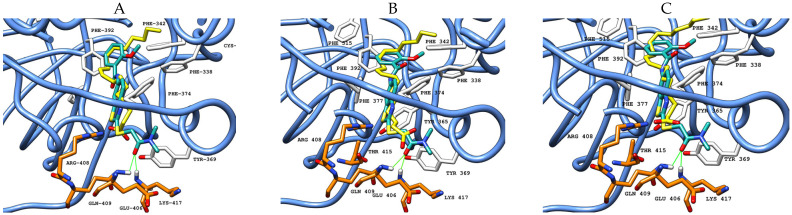
Molecular docking results obtained for **20** (carbon atoms in sea green) in the FABP of SARS-CoV-2 S protein Wuhan (PDB ID: 6ZB5, blue ribbon). (**A**) site 1 (RBD-B with carbon atoms in white, RBD-A with carbon atoms in orange); (**B**) site 2 (RBD-A with carbon atoms in white, RBD-C with carbon atoms in orange); (**C**) site 3 (RBD-C with carbon atoms in white, RBD-B with carbon atoms in orange). Green solid lines represent polar interactions (hydrogen bonds) between the ligand and amino acid residues in the protein.

Using compound **20** as an example, binding is shown in the three FABP sites (Figure 6), consistent both regarding pocket occupation and main interaction residues.

### 2.2. In Vitro Validation Assays

**Figure 7:**
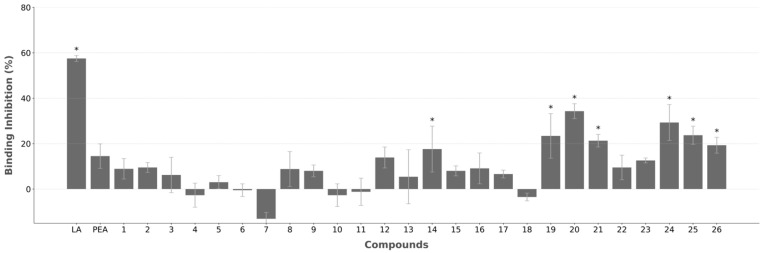
Inhibitory activity of the screened compounds tested at 100 μM. Inhibition was determined as percentage calculated on the vehicle-treated cells (0.1% DMSO). The bars represent the mean ± SEM from three experimental repeats. *—Compounds with higher activity than PEA.

The identified compounds’ ability to block the virus–host interaction was tested using an ELISA-based inhibition assay, at 100 μM, with activity compared with negative control (vehicle—1% dimethyl sulfoxide (DMSO)). LA and palmitoylethanolamide (PEA) were used as positive controls for inhibitory effect at 100 μM, given that activity has been established for these compounds: LA has been shown to reduce RBD binding with ACE2 by 100% at 8.9 mM, and PEA reached ~50% inhibition of this interaction at 10 μM [23,24]. At 100 μM, test compounds inhibitory effect ranged from 0% to 34%, with seven compounds surpassing PEA activity (14%), although LA showed the strongest inhibitory activity (58%) (Figure 7).

### 2.3. Cell Viability

**Figure 8:**
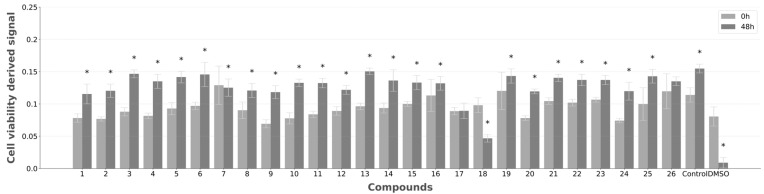
Cytotoxicity of the screened compounds at 100 μM, measured with Presto Blue^TM^ Viability Assay. Cytotoxicity variation at timepoints 4 h and 48 h was estimated against an initial measurement (0 h) before presence of screened compounds. The bars represent the mean ± SEM from three experimental repeats. *—Statistical significance difference between cell viability at both timepoints for each compound, determined by T-test for independent samples (*p* < 0.05).

A cytotoxicity evaluation of the 26 compounds was performed in L929 cells, fibroblastic-like cells extracted from mouse (Mus musculus) subcutaneous areolar and adipose tissue. This immortalized cell line provides fast, consistent, and uniform growth, facilitating acute and hyperacute toxicity testing, with high sensitivity to low concentrations. It is easy to culture and adapt to various conditions, widely used, and with predictive value validated in the literature [25]. Cytotoxicity was performed at 100 μM, with two timepoints established and cell viability measured at 0 h and 48 h (Figure 8). In the virtual screening selection protocol, predicted toxicity was an important consideration, with ADME and PAINS (pan-assay interference compounds) analysis included to exclude potential toxic compounds (Supplementary Data file) [26].

Cell viability was measured with the Presto Blue^TM^ viability assay method (Figure 8) [27]. In addition to the test compounds, two additional sets were tested, cells exposed only to growth medium (negative control), and cells treated with DMSO (positive control), since this compound is capable of altering cell membrane permeability and selectivity, justifying its use as evidence of cytotoxicity [28].

As observed in the negative control, in cells that were only exposed to growth medium (control), an increase in cell viability is reflected in a stronger signal detected. On the other hand, DMSO-treated cells, acting as a cytotoxicity control, showed a stark decrease at 48 h, due to the expected cytotoxic effect of this substance. Apart from DMSO, compounds **7** and **18** showed a reduction in cell viability at this timepoint, while **17** and **26** showed a stabilization in cell growth (non-statistically significant differences (T-test for independent samples)). Each test group showed a statistically significant cell viability variation against DMSO-treated cells (one-way Anova with Dunnett’s post hoc testing), as none of the compounds had a strong cytotoxic effect.

## 3. Discussion

From the initial set of 26 compounds, seven have shown the ability to affect the interaction more than PEA (14%), while none surpassed LA with 58% inhibitory activity. The maximum detected inhibition was 34% for compound **20**, with **14**, **19**, **21**, **24**, **25**, and **26** also surpassing PEA. Given that PEA has shown the ability to affect the virus life cycle through a reduction in the S protein–ACE2 interaction, the screened compounds might also harbor the potential to replicate this activity. In the presence of LA, the S protein is stabilized in an inactive conformation, with RBMs hidden, and the ability to interact with the human receptor severely reduced [15]. The previously reported inhibitory activity of LA was observed in these binding assays and, although the screened compounds showed weaker effects, they likely can affect the S protein behavior through the FABP. Additionally, the large variations in activity between compounds show a link to structural differences.

Overall, in the screened compounds, and unlike LA, large substituent groups are present in the deeper areas of the pocket, with aromatic rings deviating from the alkane chains in co-crystalized LA, potentially promoting aromatic interactions. This is particularly significant for **25**, where a six-membered ring with multiple heteroatoms shows pocket occupation similar to LA. Despite the fact that this large ring system is not aromatic, there are two carbonyl groups aligned with phenylalanine residues, overlapping the LA unsaturation, potentially establishing the similar stabilizing interactions. A carboxylic acid, as with LA, establishes a single H-bond with Arg408. While this compound shows some activity, the carboxylic acid is not the only terminal hydrophilic group in the active compounds (Figure 5D). The most active among screened compounds (LA achieves the highest activity), **20,** has a terminal amide group in this area, predicted to establish two H-bonds, as opposed to a single H-bond for LA, suggesting that it could be well suited to interact in this area.

However, the single H-bond established by LA is a charged H-bond, while this is not observed with the terminal amide group in **20**. Regarding the deeper areas of the pocket, while it is not as buried as LA, the terminal ring is predicted to overlap the unsaturation in LA. These two factors are likely the reason it was the best among the screened compounds, while still lacking when compared with LA (Figure 5B). Compound **24** has a nitro group, predicted as an H-bond acceptor, that extends to the hydrophilic area, and an H-bond predicted with the indole group (Figure 5C). Compound **19** has a bicyclic ring on both ends of the molecule, and groups capable of interactions in the intermediate portion of the FABP, adopting a conformation similar to LA (Figure 5A). However, **19** is predicted to establish a single H-bonds with Arg408, from the carbonyl group.

While the scoring functions and consensus protocol prioritized compounds based on predicted binding affinity, the ability to effectively engage with all three pockets served as an additional distinguishing factor. The pockets share the same amino acid composition, establishing similar interactions. Identifying compounds that exhibit strong and consistent binding across all three pockets was essential to validate the overall effectiveness of the virtual screening. Compound **20**, the strongest among screened compounds, adopts a similar conformation in all pockets, as it establishes the same H-bonds, showing the ability to maintain key hydrogen bonds across all pockets (Figure 6).

**Figure 9:**
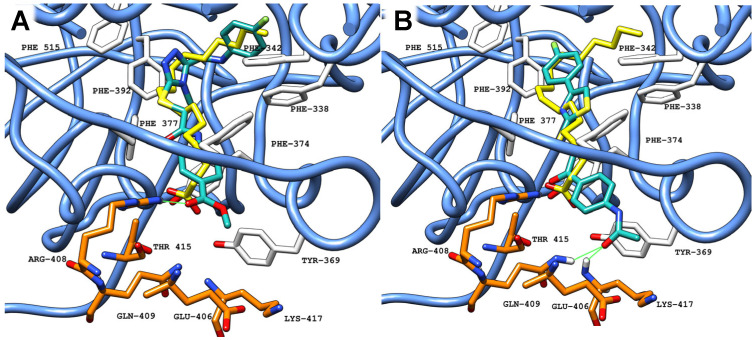
Molecular docking results obtained for **22** ((**A**), carbon atoms in sea green) and **23** ((**B**), carbon atoms in sea green) in the FABP of SARS-CoV-2 S protein (PDB ID: 6ZB5, blue ribbon and carbon atoms in white).

Finally, the detected inhibitory activity observed for **22** and **23**, although limited (9% and 13%, respectively), could be associated with a feature unique to these compounds, among the screened selection. They both share a 4-fluorophenyl entity that is predicted to extend into the FABP and likely establishes aromatic interactions in this area, particularly with Phe338, Phe374, and Phe392, although **22** is predicted to be further buried in this area and to have significant overlap with LA (Figure 9).

Furthermore, both compounds contain polar groups, such as an amide (**22**) and ketone (**23**), in the middle portion of the molecule, potentially capable of interacting with Tyr365 and Tyr369. An amide (**23**) and a methyl ester (**22**) are responsible for the stabilization in the pocket entrance, having predicted H-bonds with Gln409, Arg408, and Lys417.

Although strong cytotoxicity was not observed for any of the test compounds, **7** and **17** displayed weak cytotoxicity, maintaining the cell viability levels from the earlier timepoint (Figure 8). Cell viability for **18** had a strong reduction, although it did not reach the cytotoxicity levels showed by DMSO. During the virtual screening study, compound selection was performed considering toxicity predictions with the SWISS-ADME tool, and therefore strong cytotoxicity was less likely to arise [26]. This tool considers two complementary methods for pattern recognition, considering fragments with potential for cytotoxicity: pan assay interference compounds (PAINS), which show potent biological response irrespective of intended target, and Brenk et al.’s list of fragments or compounds that are putatively toxic, chemically reactive, metabolically unstable, or bear properties responsible for poor pharmacokinetics [26]. PAINS and Brenk warnings were, therefore, few by design, with only **21** showing a Brenk warning (Michael acceptor group) among the compounds showing cytotoxicity.

Overall, the initial virtual screening study targeting the S protein identified several compounds with activity against the virus–host interaction, reaching an inhibition of 34%, with no toxicity observed after 4 8 h. The reliability of computational methods, particularly virtual screening, is also observed in this study since, from an initial set of ~350,000 compounds, the selected molecules submitted to validatory experimental assays resulted in four compounds with inhibitory activity of the target interaction over 25%. The observed effect against the S protein could be, at least partially, due to interactions in the FABP, stabilizing the inactive protein conformation, reducing binding between ACE2 and the S protein. Cytotoxicity studies provided important insights to exclude toxic compounds, aiding the selection of the best candidate molecules for follow-up studies. The next stage for compound activity validation could involve conducting cell-based infection assays to assess antiviral effect and cytotoxicity, evaluating the ability to affect the virus–host interaction. This would be reflected in a reduced viral effect and increased cell viability due to reduced cell recognition and infection.

## 4. Materials and Methods

### 4.1. Virtual Screening

A library of commercially available drug candidates, the SPECS library, was screened against the fatty acid binding pocket using co-crystallized LA in the S protein (downloaded from the protein data bank PDB (http://www.rcsb.org/ (accessed on 15 May 2023); PDB ID: 6ZB5). The structures of the compounds analyzed were built in MOE2019.10, saved in .sdf format, and prepared using the Maestro LigPrep tool by energy minimizing the structures (OPLS_2005 force field) and generating possible ionization states at pH 7 ± 2, tautomers, all possible stereoisomers per ligand, and low-energy ring conformers. The protein was pre-processed with the MOE Protein Preparation tool, and the resulting protein–ligand complex was saved in .mae format and prepared using the Schrödinger Protein Preparation Wizard by assigning bond orders, adding hydrogens, and performing a restrained energy minimization of the added hydrogens using the OPLS_2005 force field. Additionally, the protein was also saved in .oedu format and .mol2 format to be used with scoring software CHEMPLP (PLANTS) 1.2 and ScorePose (OpenEye) 2.8.27.82.48.74, respectively [20,21]. The Glide Standard Precision virtual screening tool (SP) was used to virtually screen the commercial database against the binding site [15]. A 15 Å docking grid was prepared using the co-crystallized LA as the centroid, in parallel with the three FABPs in the S protein. The library was docked on the active sites using the Glide SP docking algorithm [18], keeping the default parameters, setting to three the number of output poses per input ligand to include in the solution, and performing a post-docking minimization of each of the poses kept. The output poses were saved as mol2 files. Docking poses obtained were then rescored (maintaining the identified pose) using Glide XP, CHEMPLP (PLANTS), and OpenEye (ScorePose) scoring functions [19,20,21]. Using a single docking program and scoring function might introduce potential bias, which justifies the use of three programs for rescoring. The values of each scoring function for each docking pose were then combined (consensus score) and only docking poses falling in the top 25% of the score value range for all the three scoring functions were selected for visual inspection in the three FABPs. The docking scores are reported in the Supplementary Data File (Tables S2–S4). The docking results were visually inspected in MOE 2022.02. The docking poses of the compounds obtained from the visual inspection were evaluated considering the following criteria: ability of a compound to adequately occupy the fatty acid binding site (similar to LA); and interactions predicted between compound and protein residues defining the site. Given that LA has been confirmed as a ligand and has shown antiviral activity in experimental assays, docked molecules were superimposed with a crystallographic structure of LA bound to the S protein (PDB accession code 6ZB5). In the next step, the set of molecules from each pocket was combined, with only molecules capable of good, predicted interactions in all pockets selected for further stages. Finally, the set of molecules identified for experimental validation was reduced to 26, by applying the Lipinski rule of five (selecting for good medicinal chemistry properties) and the SWISS-ADME webtool, to screen compound potential for toxicity (PAINS and BREAK analysis) [26].

### 4.2. Source of Small Molecules

All the compounds in this study, both the initial screened set and analogue compounds, were purchased from Specs Compound Handling B.V (Zoetermeer, the Netherlands). The library used for the virtual screening study was based on the Specs collection of screening compounds [29]. Molecular formulas (SMILES), molecular weight, and PAINS and BRENK analysis of tested compounds are reported in the Supplementary Data file.

### 4.3. Binding Assays

An Inhibitor Screening Assay Kit was used to screen inhibitors of the S–ACE2 interaction (BPS Bioscience Catalog # 78012) [30]. The kit includes the S protein in its native trimeric conformation from the Wuhan strain, providing the best physiologically relevant model for this interaction [31]. The assay kit also contains Biotinylated-ACE2, Streptavidin-HRP, and assay buffers. The assay procedure was performed as follows: SARS-CoV-2 S protein was first coated onto a 96-well plate. Following this, Biotinylated-ACE2 was incubated with the S protein on the plate, and Streptavidin-HRP was added to the plate. The interaction between Biotinylated-ACE2 and SARS-CoV-2 S protein was then detected using a colorimetric substrate. The resulting color change was quantified by measuring the absorbance using a UV–Vis microplate reader. Compounds were dissolved in DMSO and diluted until a testing concentration of 200 μM, with each compound tested in triplicate. Finally, one negative control (vehicle–1% DMSO) and two positive controls were used, LA and PEA.

### 4.4. Cell Culture

L929 cells (a fibroblastic-like cell line derived from mouse connective tissue) used for determination of cytotoxicity were cultivated in Dulbecco’s Modified Eagle Medium (DMEM) with 10% FBS, 1.5% penicillin G/streptomycin (P/S), and maintained in standard culture conditions, namely, in a humidified incubator at 37° and 5% CO_2_. Cells were evaluated daily, and the culture medium was changed whenever necessary, with cell passaging performed when desirable confluences of 70–80% were observed.

### 4.5. Cytotoxicity—Presto Blue^TM^ Viability Assay

The Presto Blue™ assay was performed to determine the cytocompatibility between the cellular system and test compounds [25,27]. This assay is based on a ready-to-use, commercially available water-soluble preparation and allows a live-cell evaluation. The resazurin solution was used to assess cell viability, based on the mitochondrial metabolization of this substance solution. Viable cells reduce the phenoxazine dye (resazurin), which results in color modification from blue to reddish over time that can be not only directly observed but also quantitively measured using UV–VIS spectrophotometry, functioning as a cell viability indicator. L929 cells were seeded over a 96-well plate and maintained in incubation overnight (standard culture medium, 37 °C, 5% CO_2_ environment, and 80% humidified atmosphere). To perform the Presto Blue™ assay, the culture medium was removed from each well at every timepoint (24, 28, and 96 h) and replaced by complete medium with 10% (*v*/*v*) of 10 Presto Blue™ cell viability reagent (Invitrogen, A13262, Thermo Fisher Scientific, Waltham, MA, USA). To perform the analysis, cells were incubated for 60 min at standard conditions to allow metabolization of the reagent. The supernatant medium was then collected and transferred to a 96-well plate and absorbance was read at 570 nm and 595 nm in a Multiskan™ FC Microplate Photometer (Thermo Scientific™, 51119000, Thermo Fisher Scientific, Waltham, MA, USA). Afterwards, wells were washed with Dulbecco’s phosphate-buffered saline solution (DPBS, Gibco, 14190169) until the Presto Blue sediments were removed. Then, fresh culture medium was added to each well, according to the timepoint specifications. Regular growth medium was used until the first timepoint (24 h), when it was replaced with DMEM (10% FBS, 1% P/S) supplemented with the test compound (determination of acute cytotoxicity). At the second timepoint (28 h), the medium was replaced with DMEM (2% FBS, 1% P/S) supplemented with test compounds, then left for 72 h until the last timepoint (96 h) (determination of acute cytotoxicity). For the Presto Blue assessment, both control group and test compounds were considered and, for each group, blank wells (without cell seeding), were included. The wavelength for excitation is 570 nm and, for emission, 595 nm. For that reason, the value obtained at 595 nm was subtracted from the value obtained for 570 nm (normalized value), for each well. In addition, the corrected absorbance for each experimental well, only considering seeded wells, was obtained by the subtraction of blank wells average from the normalized values of the respective sample group. The absorbance values were measured in triplicates. Data were further processed and normalized to the mean of the gold standard group and presented in a ratio between the 24 h timepoint, and both the 28 h and 92 h timepoints, representing variation against initial cell viability as a baseline. Statistical analysis was performed with a one-way Anova, with Dunnett’s test post hoc.

## 5. Conclusions

SARS-CoV-2 is the third highly pathogenic coronavirus to infect humans, and although the COVID-19 pandemic has largely evolved to an endemic phase, the search for new antivirals is still relevant, both for current and potentially future coronavirus infections. Given the essential functions in the virus lifecycle, the S protein emerged as a target with strong potential. We explored a binding pocket in the S protein that stabilizes an inactive conformation, the fatty acid binding pocket, capable of affecting the virus lifecycle. Through an in silico virtual screening approach, 26 potentially active compounds were selected for experimental validation. Binding assays targeting the spike–ACE2 interaction revealed four compounds with activity over 25% and low cytotoxicity at 100 μM. Further experimental testing, particularly cell-based infection assays, should be conducted to clarify the ability of the molecules to affect the virus lifecycle, derived from inhibitory activity in the virus–host interaction. These results highlight the reliability of computational techniques for discovering novel scaffolds and potentially bioactive compounds against a predetermined target in a rational methodology in drug discovery.

## Data Availability

Data are contained within the article and Supplementary Materials.

## Supplementary Materials

The following supporting information can be downloaded at: https://www.mdpi.com/article/10.3390/ijms26010151/s1.
