# Supplementary material for: Disrupting SARS-CoV-2 Spike Protein Activity: A Virtual Screening and Binding Assay Study
Source: Int J Mol Sci. 2024 Dec 27;26(1):151. doi: 10.3390/ijms26010151 (PMC11720127; doi:10.3390/ijms26010151)
Supplement: Supplementary file 1 [file ijms-26-00151-s001.zip › ijms-3304753-supplementary.pdf]

International Journal of *Molecular Sciences*

Article

Disrupting SARS-CoV-2 Spike Protein Activity: A Virtual Screening and Binding Assay Study

Luís Queirós-Reis[1]*, Rui Alvites[1,2,3,4], Ana Colette Maurício[1,2,3], Andrea Brancale[5], Marcella Bassetto[6,7],

João R. Mesquita [1,8]

[1] Abel Salazar Institute of Biomedical Sciences (ICBAS), University of Porto, 4050-313 Porto, Portugal; up201205115@up.pt (L.Q.-R.)
[2] Animal Science Study Centre (CECA), University of Porto Agroenvironment, Technologies and Sciences Institute (ICETA), 4051-401 Porto, Portugal
[3] Associate Laboratory for Animal and Veterinary Science (AL4AnimalS), 1300-477 Lisboa, Portugal
[4] University Institute of Health Sciences (CESPU), Avenida Central de Gandra 1317, 4585-116 Gandra, Portugal
[5] University of Chemistry and Technology, Prague 166 28 Praha, Czechia; andrea.brancale@vscht.cz
[6] School of Pharmacy and Pharmaceutical Sciences, College of Biomedical and Life Sciences, Cardiff University, Cardiff, CF10 3BN, UK; bassettom1@cardiff.ac.uk
[7] Department of Chemistry, Faculty of Science and Engineering, Swansea University, Swansea, SA2 8PP, UK
[8] Epidemiology Research Unit (EPIunit), Institute of Public Health, University of Porto, 4050-091 Porto, Portugal

Academic Editor: Vincenzo Bramanti

Received: 24 October 2024
Revised: 19 December 2024
Accepted: 21 December 2024
Published: 27 December 2024

Citation: Queirós-Reis, L.; Alvites, R.; Maurício, A.C.; Brancale, A.; Bassetto, M.; Mesquita, J.R. Disrupting SARS-CoV-2 Spike Protein Activity: A Virtual Screening and Binding Assay Study. *Int. J. Mol. Sci.* **2024**, *26*, x. https://doi.org/10.3390/xxxxx

Copyright: © 2024 by the authors. Submitted for possible open access publication under the terms and conditions of the Creative Commons Attribution (CC BY) license (https://creativecommons.org/licenses/by/4.0/).

International Journal of *Molecular Sciences*

MDPI

Table 1 - Molecular formulas (SMILES), molecular weight, PAINS and BRENK analysis, and medicinal chemistry properties of tested compounds

Molecule	SMILES	MW	PAINS	BRENK	H-bond acceptors	H-bond donors	LogP
Linoleic acid	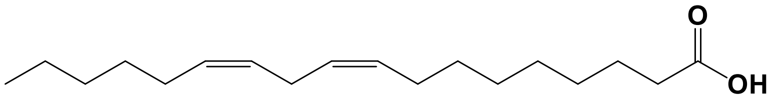	280.45	0	0	2	1	5.88
PEA	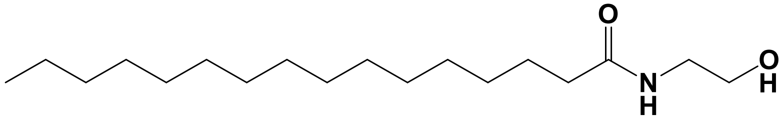	299.49	0	0	2	2	4.76
1	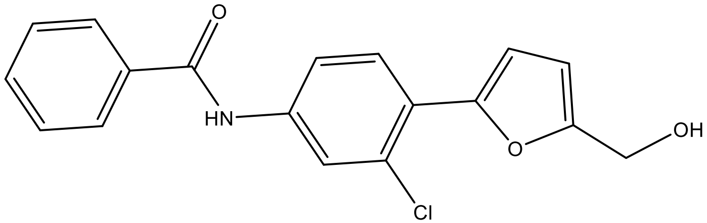	327.76	0	0	3	2	3.33
2	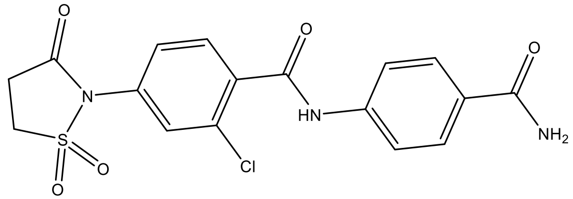	407.83	0	0	5	2	1.3
3	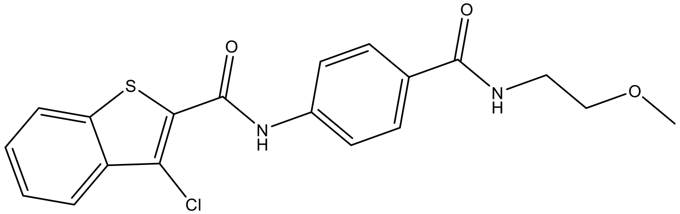	388.87	0	0	3	2	3.76

International Journal of *Molecular Sciences*

MDPI

4	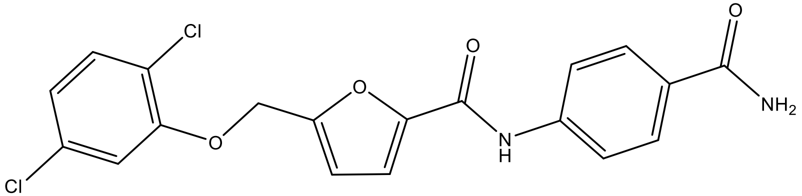	405.23	0	0	4	2	3.42
5	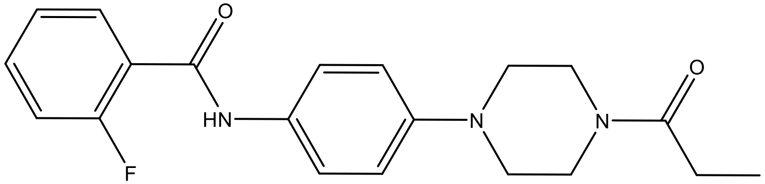	371.84	0	0	3	2	3.48
6	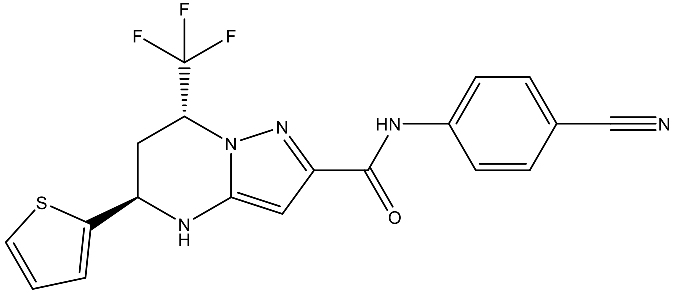	417.41	0	0	6	2	3.36
7	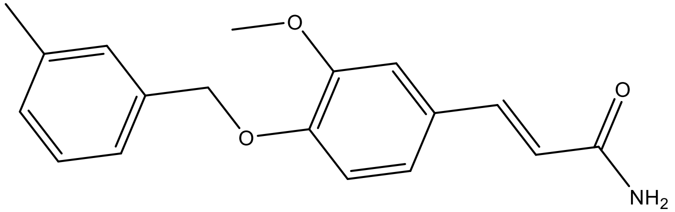	297.35	0	1	3	1	2.89
8	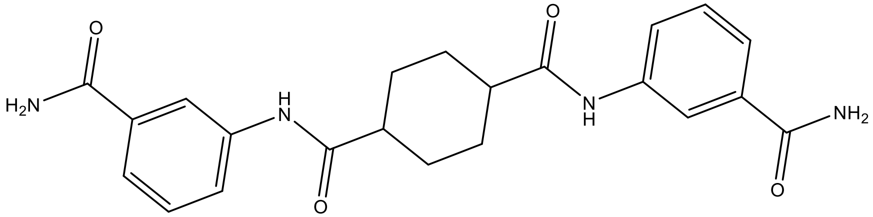	408.45	0	0	4	4	1.44

International Journal of Molecular Sciences

MDPI

9	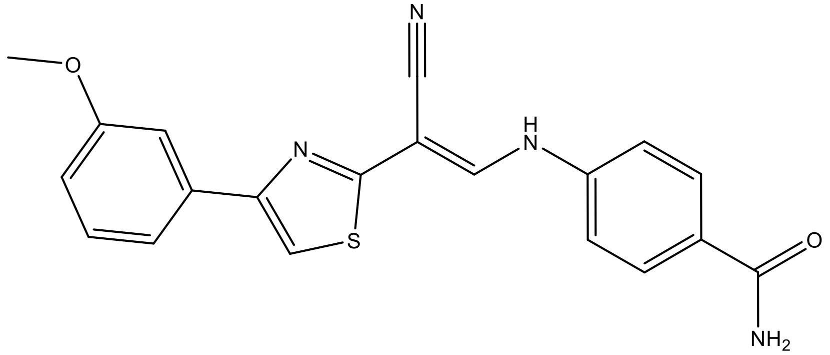	376.43	0	1	4	2	2.99
10	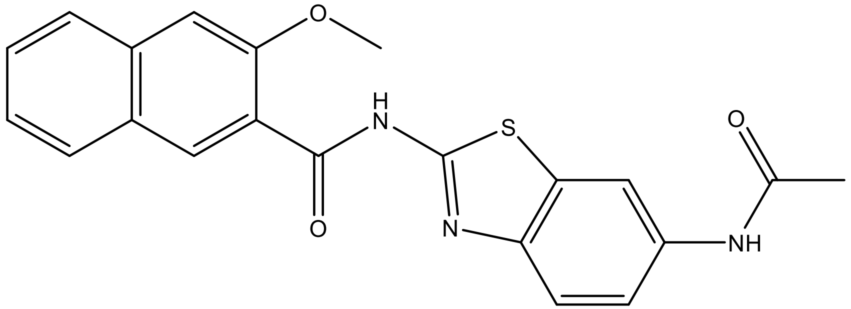	391.44	0	0	4	2	3.52
11	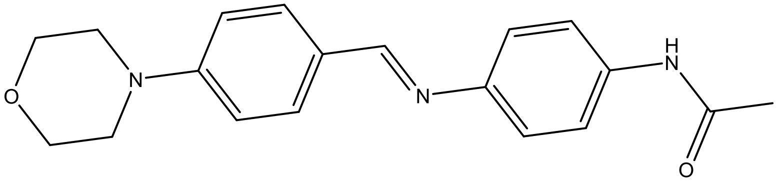	323.39	0	1	3	1	2.57
12	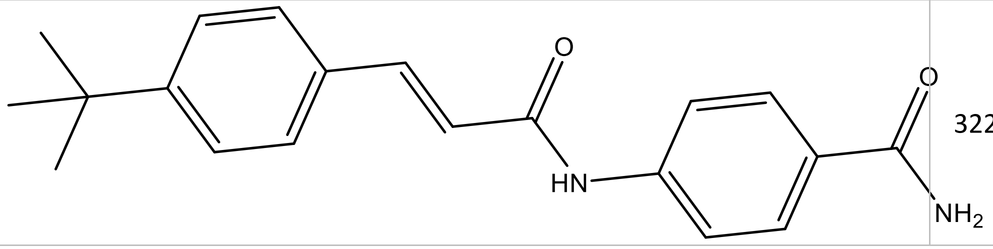	322.4	0	1	2	2	3.45

13	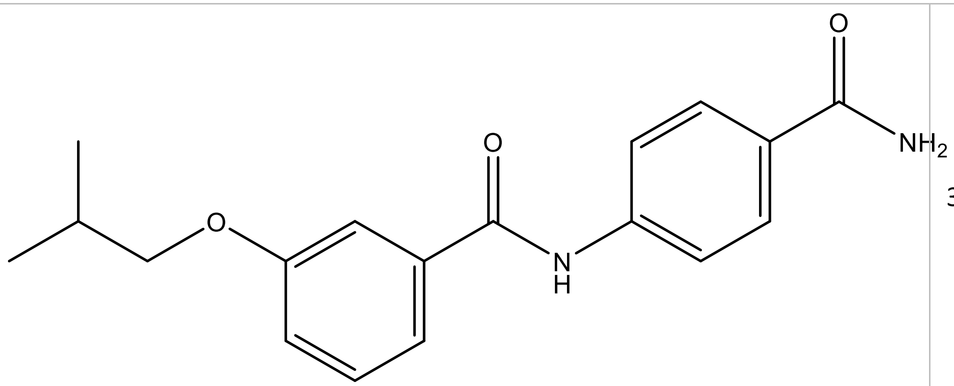	312.36	0	0	3	2	2.76
14	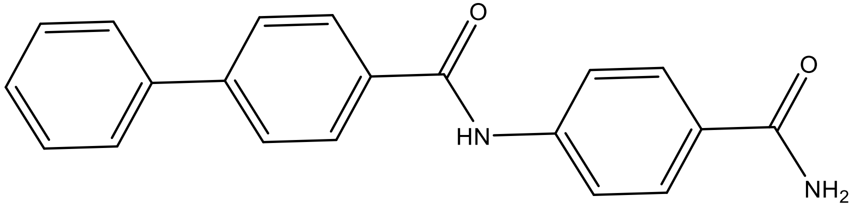	316.35	0	0	2	2	3.16
15	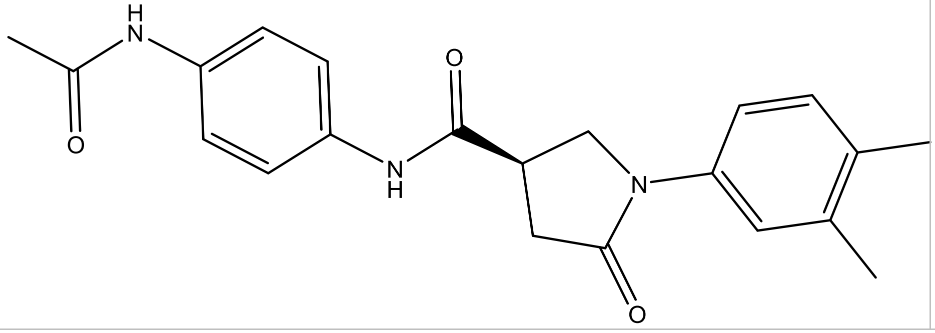	365.43	0	0	3	2	2.39

International Journal of Molecular Sciences

MDPI

16	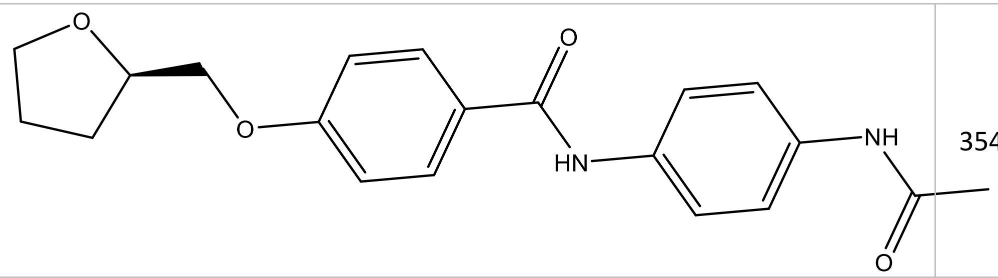	354.4	0	0	4	2	2.58
17	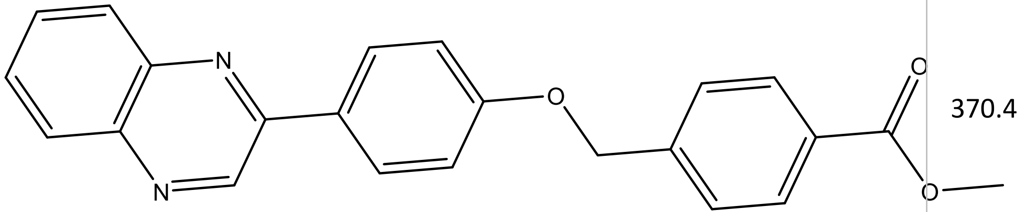	370.4	0	0	5	0	4.17
18	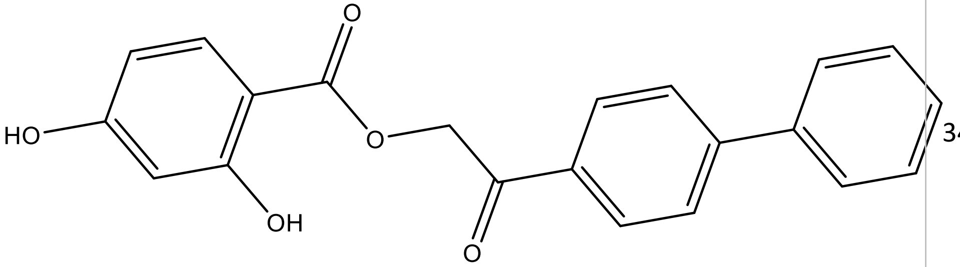	348.35	0	0	5	2	3.55
19	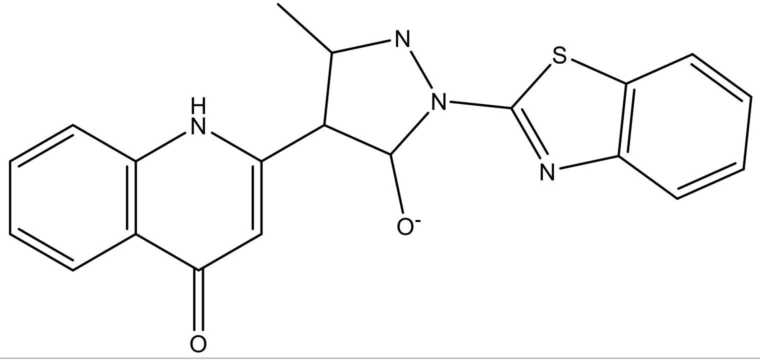	373.41	0	0	4	1	3.77

International Journal of *Molecular Sciences*

MDPI

20	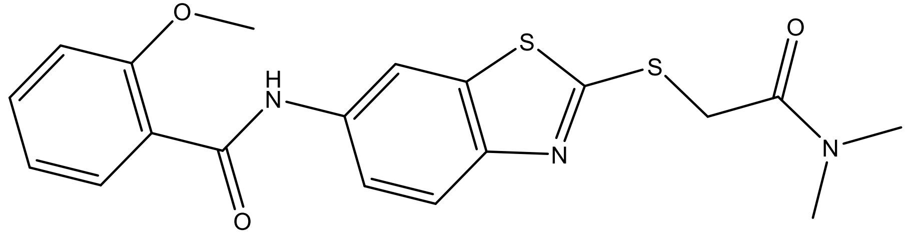	401.5	0	0	4	1	3.16
21	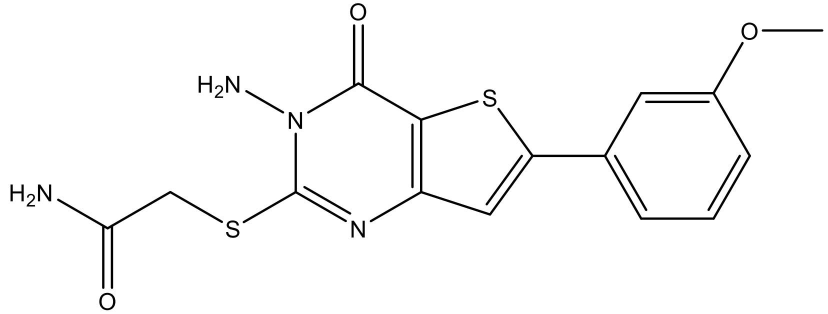	362.43	0	0	4	2	1.58
22	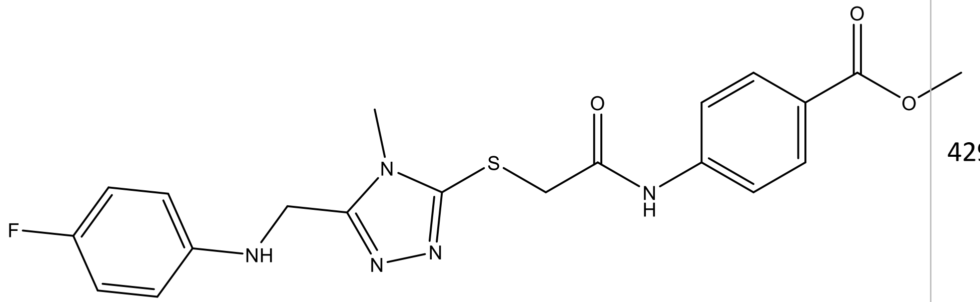	429.47	0	0	6	2	2.67
23	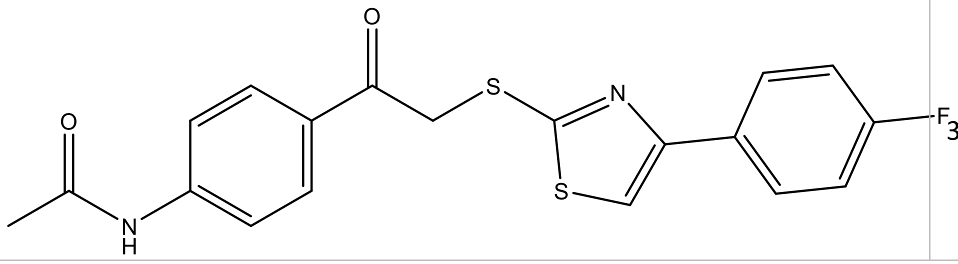	386.46	0	0	4	1	4.05

International Journal of Molecular Sciences

MDPI

24	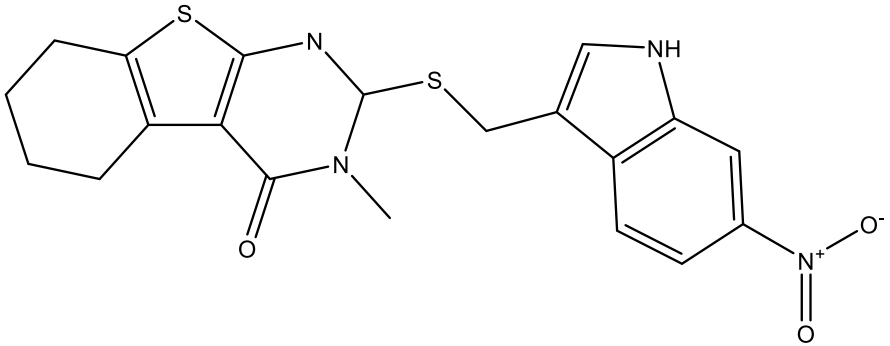	426.51	0	2	4	1	3.59
25	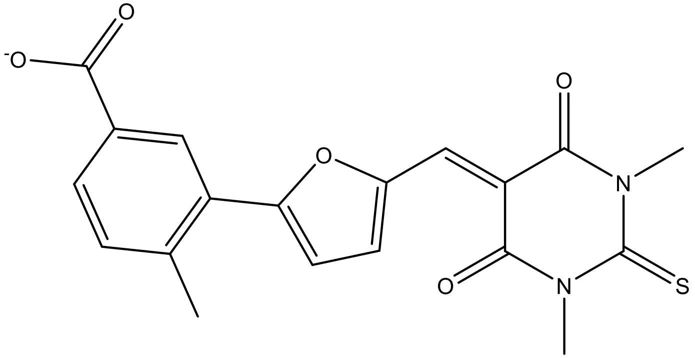	383.4	1	3	5	0	1.92
26	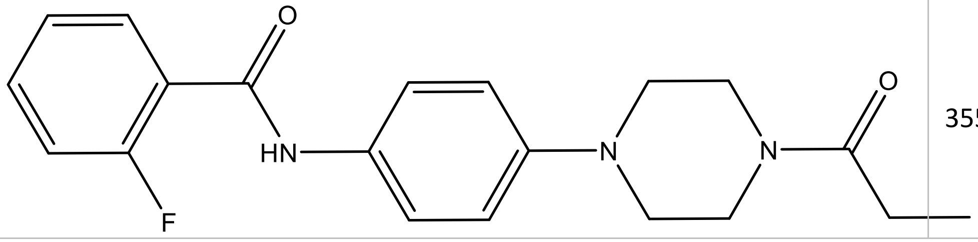	355.41	1	0	3	1	2.75

International Journal of Molecular Sciences

MDPI

Table 2 - Docking score from the scoring functions used during rescoring in FABP site 1 (Glide XP, Plants and Score Pose)

Compound	Glide XP Score	Plants Score	Score Pose Score
1	-9.90934	-19.5896	-14.2772
2	-10.8551	-19.5701	-14.1237
3	-10.4287	-18.6332	-14.3903
4	-10.2246	-19.4036	-14.3926
5	-9.63088	-19.7809	-13.6593
6	-10.5832	-19.4332	-14.5225
7	-9.56014	-19.1504	-14.2456
8	-8.71374	-18.606	-15.5326
9	-10.4791	-19.4499	-14.8625
10	-10.7591	-19.6986	-15.0075
11	-10.5762	-18.5116	-14.2431
12	-10.8036	-19.0669	-15.048
13	-10.6837	-19.6938	-13.7471
14	-10.847	-19.9558	-14.9314
15	-9.84559	-19.3128	-14.5311
16	-11.1387	-19.7257	-15.2825
17	-11.0642	-19.1502	-15.1758
18	-10.8782	-18.5412	-16.8503
19	-9.40831	-19.8365	-13.6834
20	-9.88815	-18.5055	-15.2236
21	-9.69366	-18.5623	-13.7639
22	-9.86747	-19.1916	-15.3514
23	-8.91967	-18.5418	-16.1814
24	-11.064	-19.0385	-16.1967
25	-10.6393	-19.9047	-14.7905
26	-9.48367	-20.5197	-15.7748

International Journal of Molecular Sciences

MDPI

Table 3 - Docking score from the scoring functions used during rescoring in FABP site 2 (Glide XP, Plants and Score Pose)

Compound	Glide XP Score	Plants Score	Score Pose Score
1	-9.60702	-84.0794	-14.316
2	-10.8539	-84.8198	-14.5393
3	-10.3137	-84.6865	-14.2819
4	-10.986	-96.7236	-16.302
5	-10.2643	-83.3837	-14.4076
6	-10.0921	-85.556	-15.1899
7	-9.69546	-84.4472	-14.2979
8	-10.9777	-87.697	-15.4979
9	-10.6759	-85.9965	-14.7041
10	-10.5987	-85.2048	-15.1432
11	-10.9371	-87.0268	-14.7195
12	-10.734	-84.8338	-15.2876
13	-10.5144	-87.0471	-13.7435
14	-10.6829	-88.7107	-15.1746
15	-9.49515	-84.4718	-14.8161
16	-10.9766	-93.3181	-15.1306
17	-12.254	-89.5624	-15.6337
18	-10.7716	-94.1199	-17.3446
19	-9.65984	-92.6943	-13.7081
20	-10.3088	-86.4465	-15.4393
21	-9.29881	-85.3654	-14.3168
22	-11.0059	-87.2998	-15.0335
23	-9.66039	-94.7483	-17.7919
24	-11.2866	-89.6218	-16.967
25	-10.6021	-84.3042	-15.0282
26	-9.93975	-91.2596	-16.8411

International Journal of
Molecular Sciences

MDPI

Table 4 - Docking score from the scoring functions used during rescoring in FABP site 3 (Glide XP, Plants and Score Pose)

Compound	Glide XP Score	Plants Score	Score Pose Score
1	-2.39452	-31.2631	-14.0827
2	-1.70741	-34.658	-13.7346
3	-0.793615	-35.306	-15.2858
4	-1.74717	-33.8839	-15.7387
5	-0.593194	-37.1896	-13.5115
6	-0.941544	-31.6506	-15.0051
7	-1.15977	-36.114	-13.413
8	-1.73428	-31.349	-14.6424
9	-0.766879	-34.3522	-14.6466
10	-2.10142	-35.3429	-14.6804
11	-2.85056	-32.8616	-14.3353
12	-1.71717	-34.0072	-15.2773
13	-1.87547	-33.1773	-13.7252
14	-1.97368	-34.4894	-15.0862
15	-1.15028	-33.4434	-14.0565
16	-0.93781	-33.8413	-15.1547
17	-1.79672	-36.7797	-16.7134
18	-1.24823	-36.4917	-17.4692
19	-0.986509	-44.2113	-13.449
20	-1.76133	-32.5783	-15.3749
21	-1.47461	-37.179	-13.8098
22	-0.716379	-32.5013	-14.5622
23	-1.87668	-42.4524	-15.2877
24	-0.96427	-41.0504	-16.3762
25	-0.814023	-39.0414	-14.0006
26	-0.495283	-31.3886	-15.8787
